# 4,6-Diamino-2-thiopyrimidine-based Cobalt Metal Organic Framework (Co-DAT‐MOF): green, efficient, novel and reusable nanocatalyst for synthesis of multicomponent reactions

**DOI:** 10.1038/s41598-023-34001-5

**Published:** 2023-05-09

**Authors:** Arash Ghorbani-Choghamarani, Zahra Kakakhani, Zahra Taherinia

**Affiliations:** 1 Department of Organic Chemistry, Faculty of Chemistry, Bu-Ali Sina University, Hamedan, 6517838683 Iran; 2 Department of Chemistry, Faculty of Science, Ilam University, Ilam, Iran

**Keywords:** Chemistry, Energy science and technology, Engineering, Materials science, Nanoscience and technology

## Abstract

In this study, Co-DAT‐MOF powder was prepared via the solvothermal method using 4, 6-diamino-2-thiopyrimidine as the organic linker and Co(NO_3_)_2_·6H_2_O. The synthesized catalysts are characterized using XRD, FT-IR, TGA, SEM, BET, NH_3_-TPD, and ICP-OES techniques. SEM analysis clearly indicated the formation of nanosheet microspheres. NH_3_-TPD-MS was employed as a means of identifying the various strengths of acid sites and their relative abundance in an attempt to explain the effect of the catalyst surface acid sites. We identified a new acidic feature in Co-DAT‐MOF catalyst, related to the presence of desorption peaks in the NH_3_-TPD profiles. The activity of Co-DAT‐MOF catalyst for the synthesis of multicomponent reactions correlates with lewis acidity. In addition, Co-DAT‐MOF exhibited excellent performance for the synthesis of pyrroloacridine-1(2H)-one and chromeno [2, 3- d] pyrimidin-8-amines, as well as good reusability and recyclability.

## Introduction

**Figure 1:**
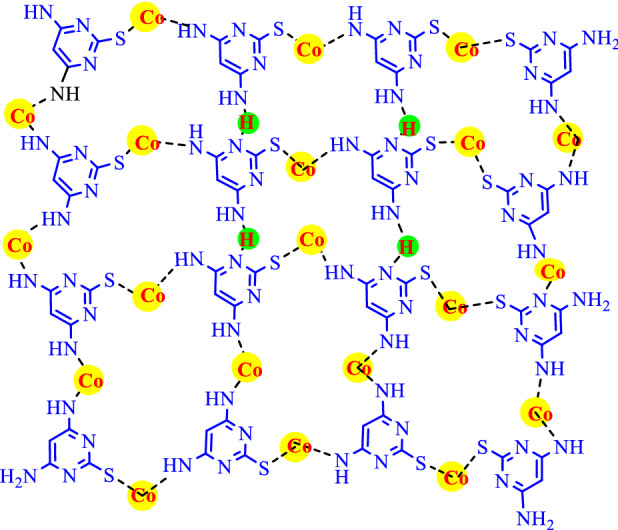
Topological structure of Co-DAT‐MOF.

So far, a wide variety of materials, as supports for solid bases, have been studied ranging from inorganic materials^1–7^ to organic ones^8^. Apparently, the support effect is of vital role in determining surface properties, activity, and selectivity of the catalysts^9^. Metal–organic frameworks (MOFs) have received a lot of attention due to their physical and chemical properties such as high surface area^10^, pore volumes^11^, high thermal and chemical stabilities^12^, and variety of pore dimensions/topologies^13^. In this sense, metal–organic frameworks (MOFs) have been widely applied in various areas i.e. catalysis^14–27^, luminescence^28^, drug delivery^29^, sensors^30^, gas storage^31^ and separation^32^. Besides, they are capable of being applied as energy storage^33^, magnetic and ion-conducting properties^34^, and conversion^35^. Multicomponent reactions (MCRs) are one-pot highly efficient transformations utilizing three or more components to afford single products with greater efficiency and atom economy^36^. Moreover, this strategy represents an efficient strategy towards sustainable synthesis*.* Recently, considerable attention has been paid toward the one-pot multicomponent reactions, due to the fact that they can be broadly applied in order to prepare the biologically relevant or natural-product-like molecular frameworks. Pyrroloacridine-1(2H)-one is an organic compound based on pyridine. Besides, this nucleus which is one of the most significant core structures can be regarded as an important part of most natural and unnatural heterocyclic compounds, has been identified as a vital drug for the treatment of cardiovascular diseases and is known as a calcium channel modulator^37^. There are different methods to synthesis acridines derivatives from dimedone, aldehyde and aniline derivatives, or ammonium acetate using various catalysts^38^. We started our study by employing isatin instead of an aldehyde with dimedone, and aniline derivatives for the synthesis of spiro[oxindole-acridine] compounds. Chromenes and their derivatives are very important heterocycles as they display a wide spectrum of biological activities; including, antimicrobial^39^, antioxidants^40^, antitumor^41^, antiproliferative^42^ and antifungal agent^43–45^. Thus*,* the development of synthetic strategies for the synthesis of molecules containing chromene and pyrimidine rings are of interest to both organic and medicinal chemists*.* In this contribution report, the synthesis of Co-DAT‐MOF was generated from the reaction of 4, 6*-*diamino-2-thiopyrimidine with Co*(*NO_3_*)*_2_*.*6H_2_O under solvothermal conditions. The obtained Co-DAT‐MOF demonstrated high catalytic activity for the synthesis of pyrroloacridine-1(2H)-one and chromeno [2, 3- d] pyrimidin-8-amines. Topological structure of Co-DAT‐MOF is shown in Fig. 1.

## Experimental section

### Synthesis of cobalt-based metal organic framework

For the preparation of Co-DAT‐MOF*,* 4, 6-Diamino-2-thiopyrimidine (1 mmol), and Co(NO_3_)_2_.6H_2_O(1 mmol) were dissolved in 20 mL of DMF and 5 mL of H_2_O. After, the sample was placed in an ultrasonic bath (2.75 L, 380/350 W, UNSPSC 42281712) filled with water by a clamp, and sonicated at room temperature and atmospheric pressure for 20 min. The ultrasonic time was 15 min, and the ultrasonic power was 100 W. Then, mixture transferred into teflon-lined stainless-steel autoclave and heated at 160 ˚C for 15 h. The precipitate was collected, washed with ethyl acetate and dried at 60 °C in the vacuum.

### General procedure for the one-pot synthesis of 7, 10-diaryl7H-benzo[7,8]chromeno[2,3d] pyrimidin -8-amine derivatives

A stirring mixture of an aldehydes (2 mmol) of α-naphthol (1 mmol), malononitrile (1 mmol), Co-DAT‐MOF (50 mg) and EtOH (5 mL) was heated under refluxing conditions in an oil bath, followed by the addition of ammonium acetate (2 mmol) at the same temperature (80 °C). Upon completion, 20 mL of ethyl acetate was added to the reaction mixture to separate the catalyst using centrifugation. The filtrate was concentrated under vacuum. The crude products were purified by recrystallization by ethyl acetate and *n-*hexane (1:6).

### General procedure for the one-pot synthesis of 4, 4-dimethyl-2-phenyl-4, 5-dihydropyrrolo[2,3,4-kl] acridin-1(2H)-one

A solution of an aniline (1 mmol), dimedone (1 mmol), isatin (1 mmol), and Co-DAT‐MOF (60 mg) in 2 mL PEG was allowed to stir at 110 °C for the appropriate time. On completion of the reaction (monitored by TLC analysis), the reaction mixture was cooled to 25 °C. At the end of reaction, 20 mL of ethyl acetate was added to the reaction mixture to separate the catalyst using centrifugation. The filtrate was concentrated under vacuum. The crude products were purified by recrystallization by ethyl acetate and *n-*hexane (1:6).

## Results and discussion

**Figure 2:**
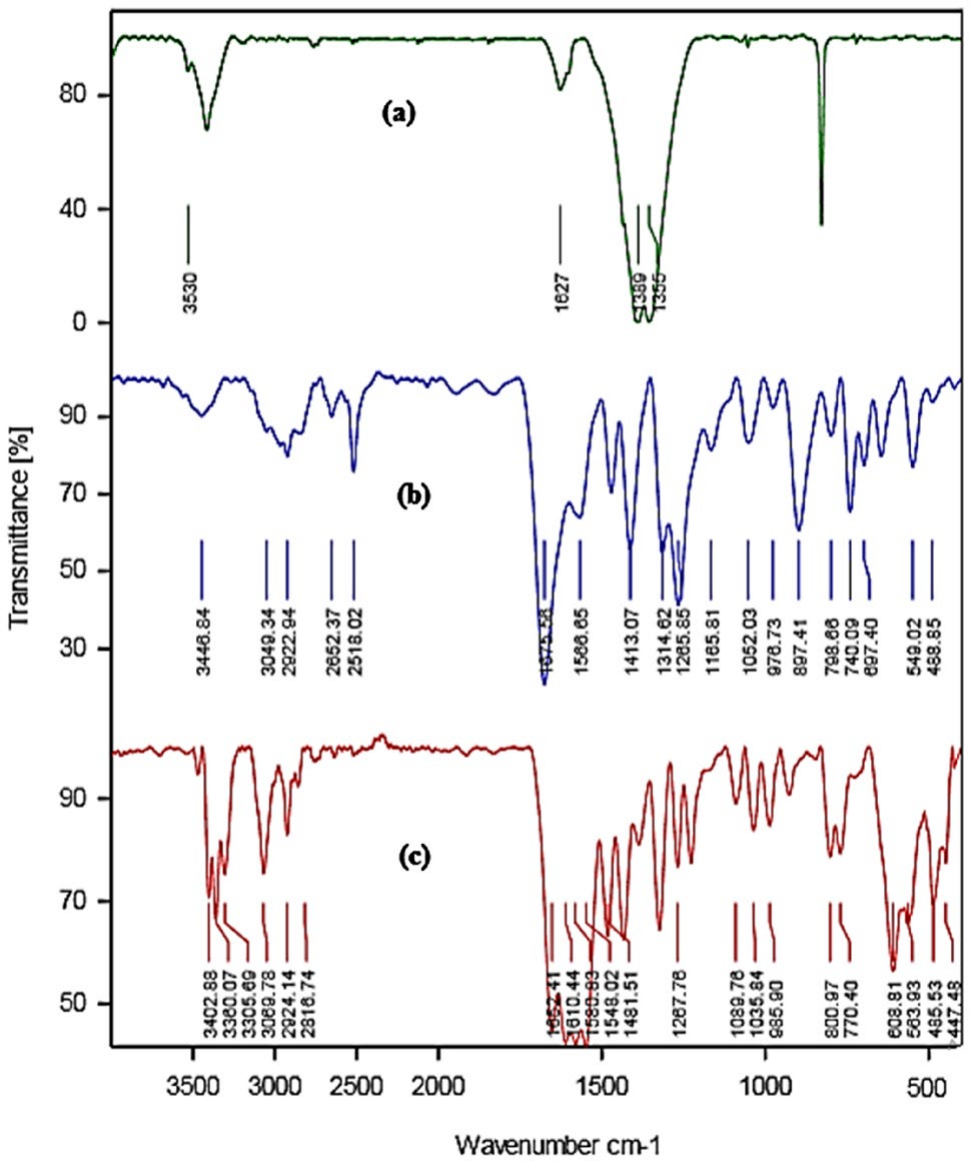
FT-IR spectra of Co (NO_3_)_2_.6H_2_O (**a**), ligand (**b**), and CoMOF (**c**).

The catalytic system has been studied by XRD, FT-IR, TGA, BET, TPD NH_3_, ICP-OES, and SEM techniques. Moreover, Fig. 2 comparatively shows the FT-IR spectra of a free ligand, Co (NO_3_)_3_.6H_2_O and Co-DAT‐MOF. In the FT-IR spectrum of free ligand(Fig. 2b), the peaks positioned at 1566, 1675, and 2518 cm^−1^ were related to stertching vibration of C = N, bending vibration of NH, and stertching band of S–H, respectively. The spectrum of the Co-DAT‐MOF, the absorption of S–H stretching band was disappeared because of the formation of Co-S bond between 4,6-Diamino-2-thiopyrimidine and Co. In addition, the bending vibration of N–H, for Co-P-MOF structure shifted to the lower wavenumber (1652 cm^−1^) which results demonstrate Co ions can be regarded as the ions modified on the surface of the ligand.

**Figure 3:**
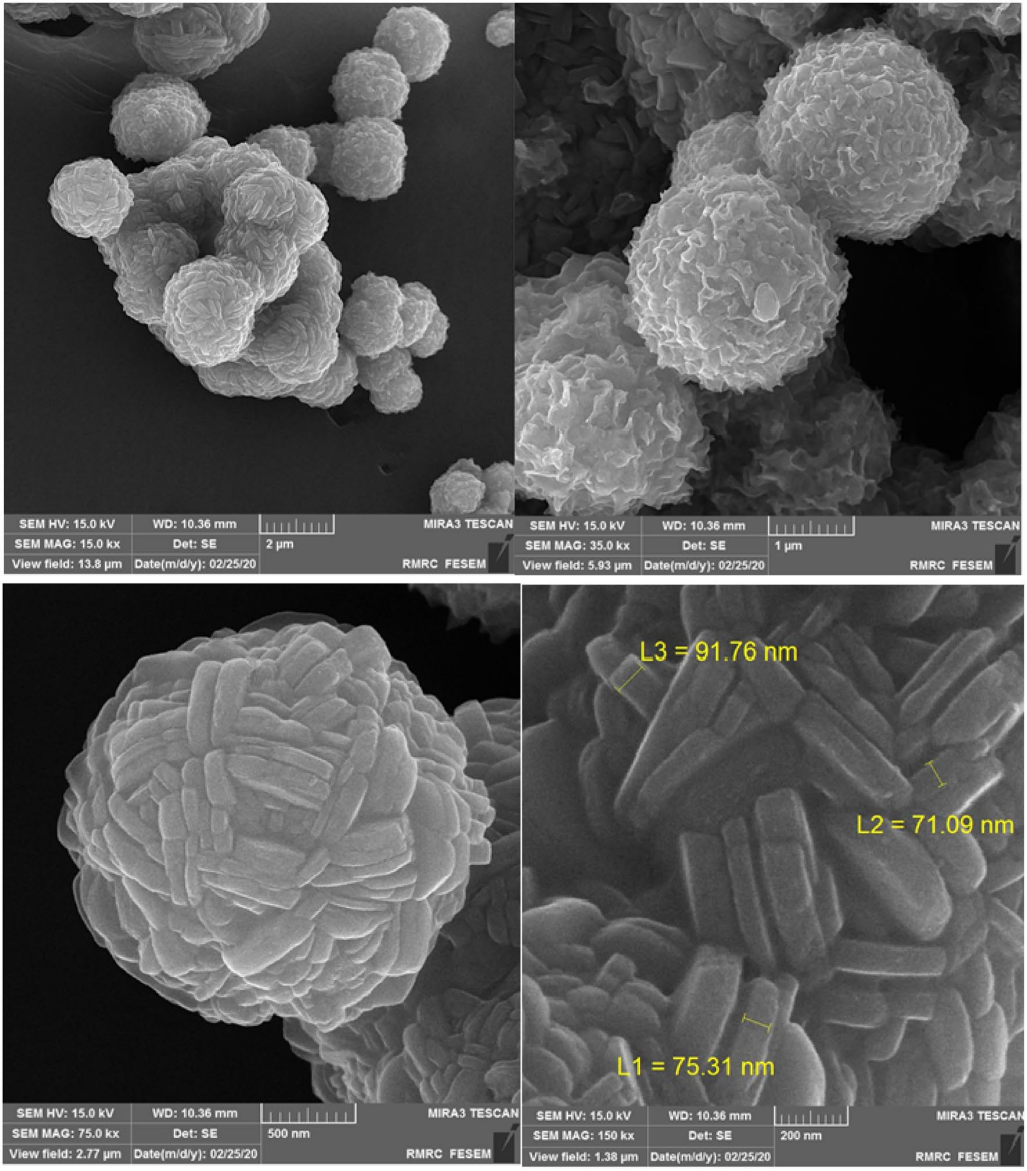
SEM images of Co-DAT‐MOF.

The overall morphology of the Co-DAT‐MOF (Fig. 3) indicates that microspheres were synthesized using this simple solvothermal method. As shown in the high-magnification SEM images, the microspheres are composed of numerous ultrathin nanosheets.

**Figure 4:**
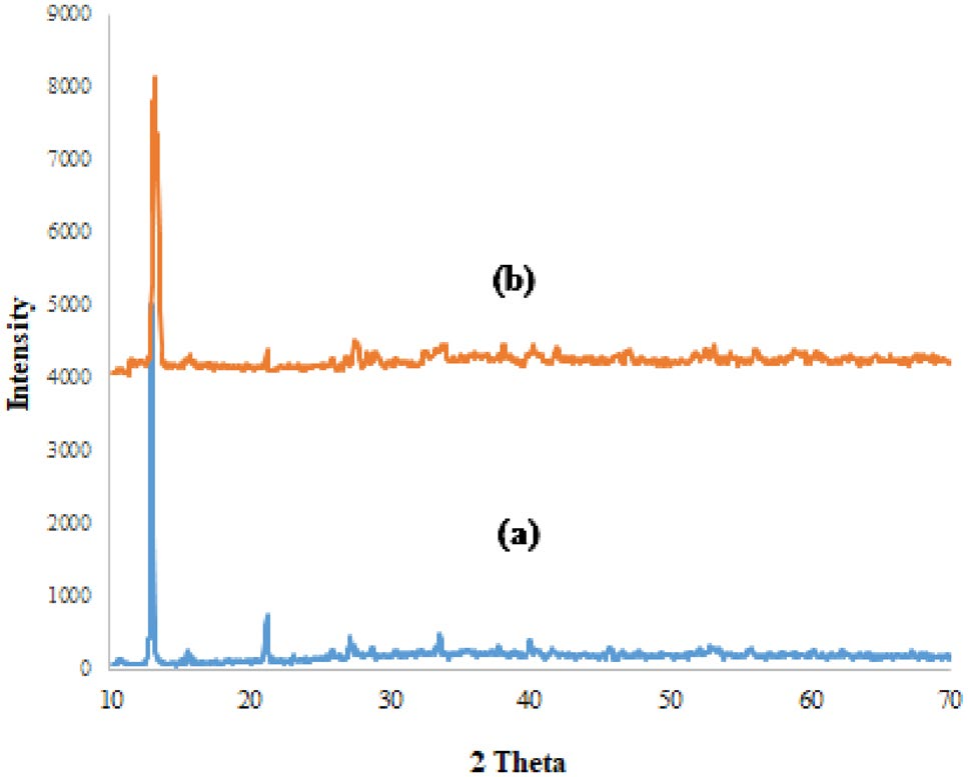
XRD pattern of Co-DAT‐MOF before recovery (**a**) after recovery (**b**).

In order to investigate the crystal structure of Co-DAT‐MOF nanocatalyst, we used XRD pattern(Fig. 4). The XRD of the Co-DAT‐MOF shows the main peak at 11.78 which corresponds to the standard pattern^46^.

**Figure 5:**
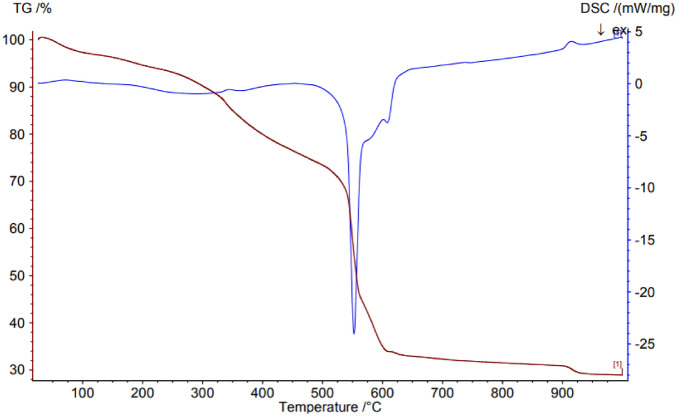
TGA thermogram of Co-DAT‐MOF.

TGA used to shows the weight loss of Co-DAT‐MOF under air atmosphere (Fig. 5). The first weight loss of 9 wt% occurs below 300 °C, corresponding to the elimination of bound DMF molecules. It also indicates a loss of one DMF molecule per host lattice. The second weight loss of 21 wt% over the temperature range of 320–550 °C can be ascribed to the decomposition of Co-DAT‐MOF and formation of cobalt oxide.

**Figure 6:**
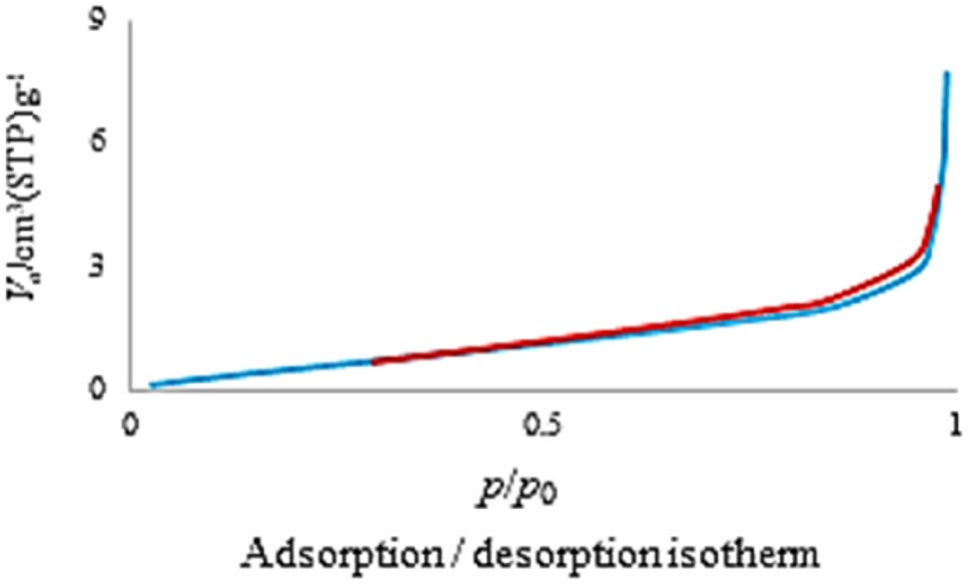
Nitrogen adsorption–desorption isotherm of Co-DAT‐MOF.

**Figure 7:**
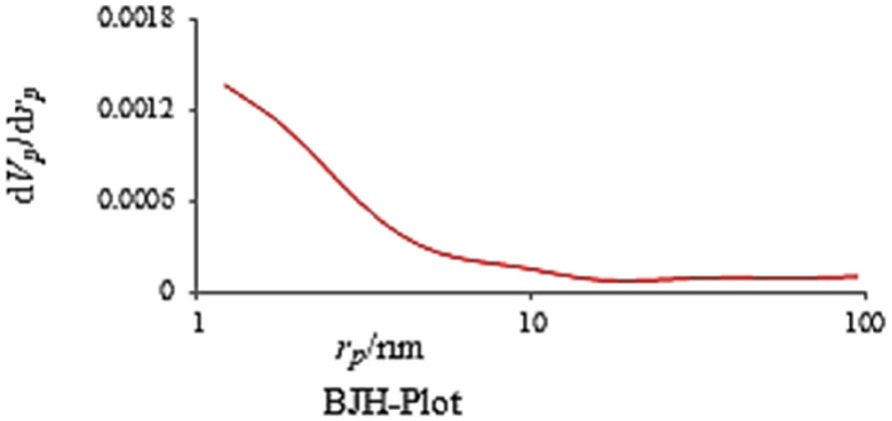
Pore size distribution curves of Co-DAT‐MOF.

The nitrogen adsorption–desorption isotherm of the Co-DAT‐MOF shown in Fig. 6 could be categorized as type IV with hysteresis loops in the range of 0.64–0.96 P/P_0_, the existence of abundant pores. The Brunauer–Emmett–Teller (BET) specific surface area was 2.5 m^2^ g^−1^. The BJH pore size calculations using the adsorption branch of the nitrogen isotherm indicate a micropore peak at about 1.66 nm for Co-DAT‐MOF (Fig. 7).

**Figure 8:**
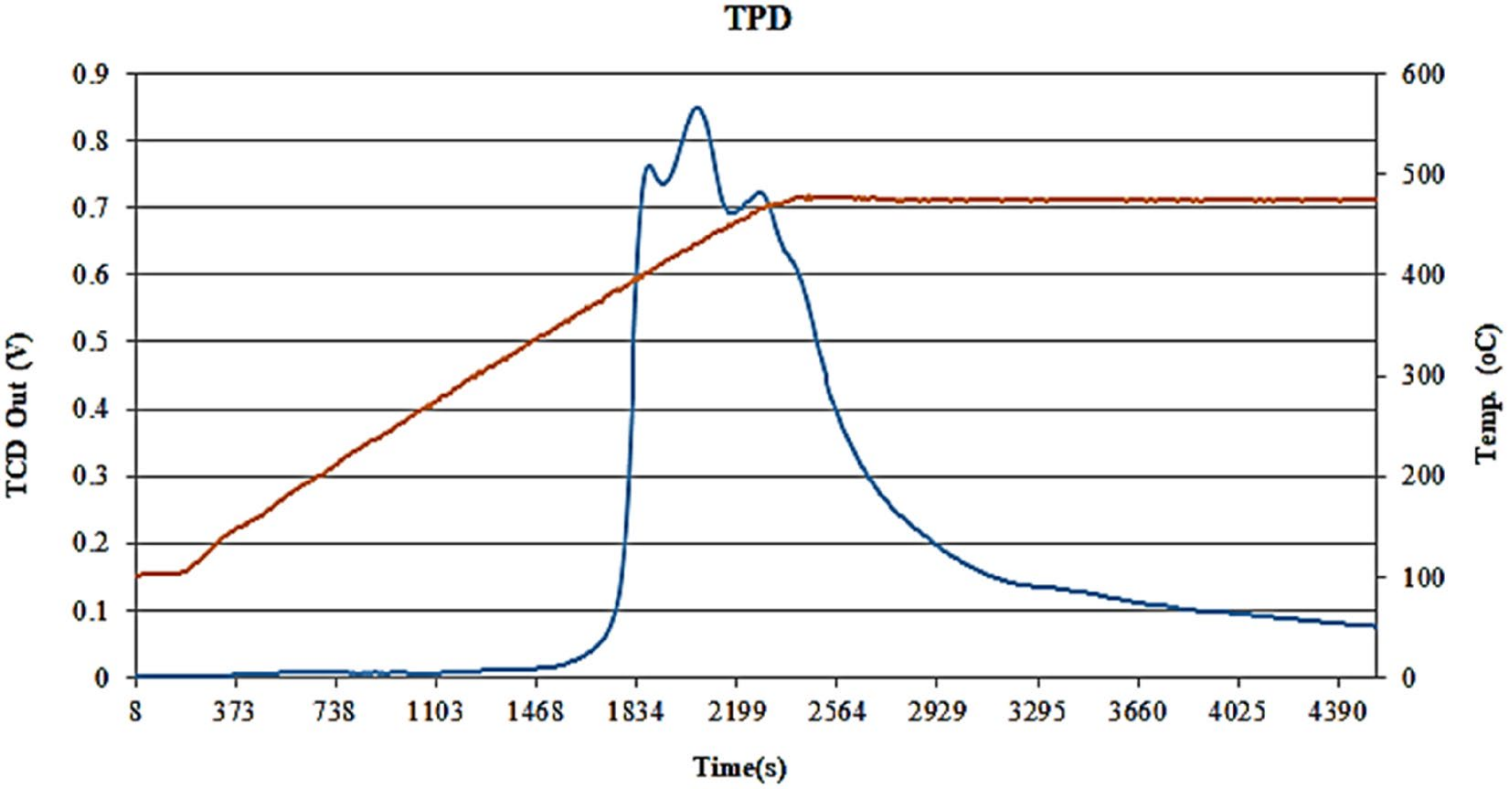
NH_3_-TPD profiles of Co-DAT‐MOF catalyst measured by NH_3_-TPD.

The NH_3_-TPD pattern of the Co-DAT‐MOF catalyst is shown in Fig. 8, Table 1 and peaks desorption of NH_3_ are observed. The peak observed at (95–300 °C) is attributed to weak acid sites and the peak at a range of temperatures (300–475 °C) to strong acidic sites. In the case of exceeding this temperature (475 °C) during the NH_3_-TPD measurements, the thermal decomposition of samples could occur and the evolved gases could be misinterpreted as ammonia because the employed detector (TCD) did not enable the identify the evolved gases.

**Table 1:** Surface acidity of Co-DAT‐MOF catalyst measured by NH_3_-TPD.

Temp. range (°C)	Area	NH_3_ desorption (micro-mol/gr catalyst)
[95–300]	5.91	36
[300–475]	815.9	4975

### Catalytic studies

**Figure 9:**
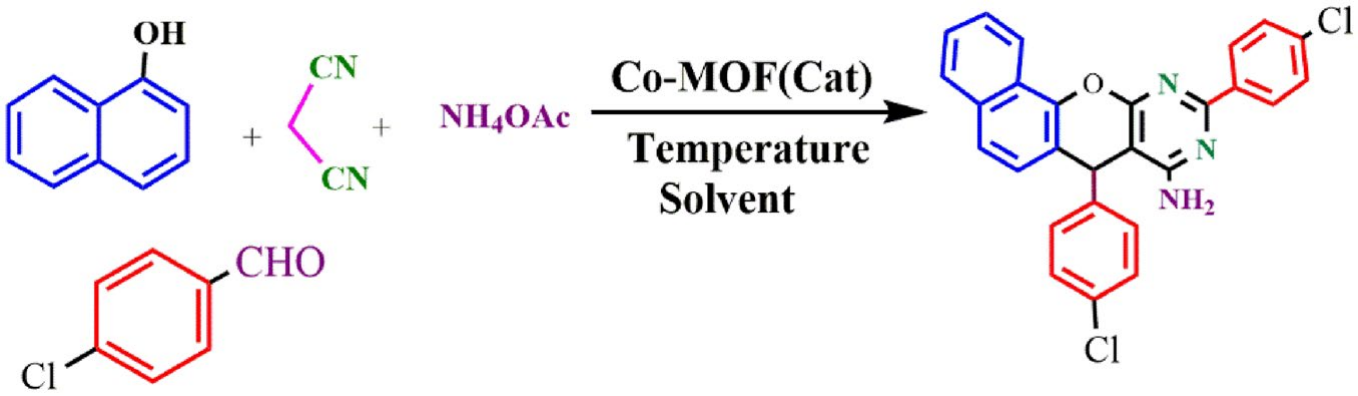
Preparation of chromeno[2,3-d] pyrimidin-8-amine.

**Figure 10:**
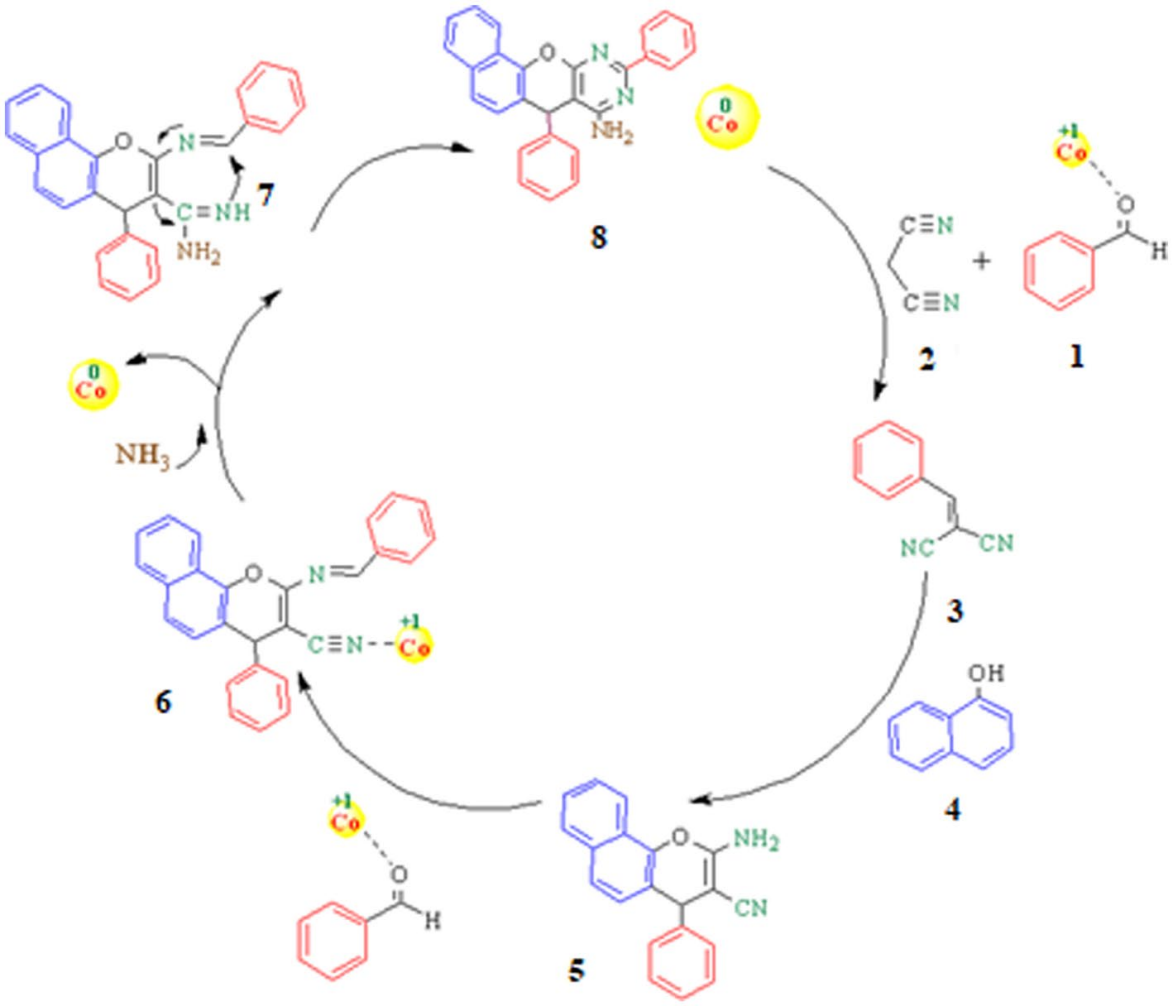
Proposed mechanism for the synthesis of 7,10-diaryl-7*H*- benzo[7,8]chromeno [2,3- d]pyrimidin-8- amine.

Applicability of Co-DAT‐MOF was investigated for the synthesis of pyrroloacridine-1(2H)-one and chromeno [2, 3- d] pyrimidin-8-amines. In the first part, a direct synthesis of Chromeno [2, 3- d] pyrimidin-8-amines via combination of, 4*-*Chlorobenzaldehyde, α-naphthol, malononitrile, and ammonium acetate in the presence of Co-DAT‐MOF was presented (Fig. 9). Initially, the effect of solvents (PEG, DMF, EtOH, H_2_O) was also investigated and it was observed that the reaction was highly effective with EtOH. Furthermore, the progress of the reaction depended on the amount of Co-DAT‐MOF; the reaction was found to complete in the presence of 50 mg of this catalyst. The control experiment confirmed that the reaction did not occur in the presence of Co (NO_3_)_3_.6H_2_O, and 4, 6-Diamino-2-thiopyrimidine as acatalyst (Table 2, entry 11–12). The reaction when conducted at room temperature and 60 °C, the yields observed were very low (Table 2, entries 5 and 6). The ideal temperature for the reaction was found to be 80 °C. Subsequently we performed the synthesis of diverse chromeno [2, 3- d] pyrimidin-8-amines with different substituted aldehyde under optimized reaction condations (Table 3). Both electron-withdrawing and electron-donating substituents on the aldehydes were found to work reasonably well, giving moderate to good yields of the final products. A plausible mechanism for the formation of chromeno [2, 3- d] pyrimidin-8-amines derivatives has been described in Fig. 10.

**Table 2:** Different reaction conditions for synthesis of chromeno[2,3-d] pyrimidin-8-amine.

Entry^[a]^	Cat (mg)	Solvent	Temp. (ºC)	Yield^[b]^
1	50	PEG	80	78
2	50	DMF	80	65
3	50	H_2_O	80	N.R
4	50	EtOH	80	98
5	50	EtOH	60	69
6	50	EtOH	25	Trace
^[c]^7	–	EtOH	80	N.R
8	20	EtOH	80	27
9	30	EtOH	80	53
10	40	EtOH	80	72
^[d]^11	50	EtOH	80	N.R
^[e]^12	50	EtOH	80	N.R

^[a]^Reaction conditions: 4*-*Chlorobenzaldehyde (2 mmol), α-naphthol (1 mmol), malononitrile (1 mmol), ammonium acetate (2 mmol), Co-DAT‐MOF, solvent.

^[b]^Isolated yield.

^[c]^When the reaction carried out in the absence of Co-DAT‐MOF.

^[d,e]^When the reaction was conducted in the presence of Co (NO_3_)_3_.6H_2_O, and 4, 6-Diamino-2-thiopyrimidine. Time: 1.5 h.

**Table 3:** The preparation of chromeno[2,3-d] pyrimidin-8-amines catalyzed Co-DAT‐MO.

Entry^[a]^	Product	Time (h)	Yield^[b]^ (%)	M.P
1		1.5	92	170–175^47^
2		1.5	93	250–260^47^
3		3	80	210–212^47^
4		2	88	162–164^47^
5		3.5	78	177–181^48^
6		1	98	192–196^47^
7		1.5	82	280–282^48^
8		1.5	75	267–270
9		1.5	96	232–234^47^
10		2.5	77	182–185

^[a]^Reaction conditions: benzaldehyde (2 mmol), α-naphthol (1 mmol), malononitrile (1 mmol), ammonium acetate (2 mmol), Co-DAT‐MOF, solvent.

^[b]^Isolated yield.

**Figure 11:**
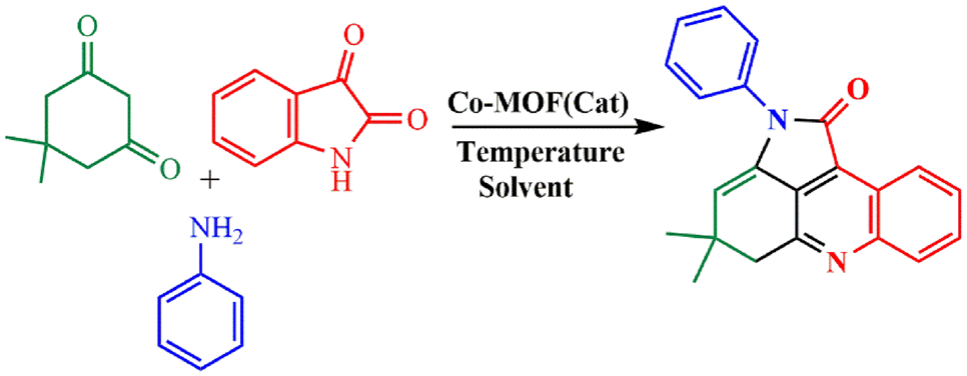
Preparation of 4,4-dimethyl-2-phenyl-4,5-dihydropyrrolo[2,3,4-kl]acridin-1(2H)-one.

The catalytic activity of Co-DAT‐MOF was evaluated for synthesis pyrroloacridine-1(2H)-one derivatives based on the one-pot three-component reaction of amine, dimedone, and isatin. We performed the reaction by conducting the reaction of aniline, dimedone and isatin as a model to optimize the process conditions in the presence of Co-DAT‐MOF (Fig. 11). We also tested the influence of solvents and found out that DMF, and PEG afforded the final products (Table 4). However, higher yields were obtained when PEG was used as the solvent. Then, the influence of temperature on the progress of the reaction was evaluated. Lower yields of the desired product was observed while decreasing the reaction temperature. Afterwards the specific amounts of the catalyst (30, 40, 50, 60 mg) were utilized in the process*.* In addition, the control experiment confirmed that the reaction did not occur in the absence of the catalyst (Table 4, entry 8). In order to broaden the scope of the developed protocol, a wide range of amines were examined for the synthesis of pyrroloacridine-1(2H)-one (Table 5). The amines containing the electron-donating as well as electron-withdrawing substituents were compatible under the optimized reaction and provided good to excellent yield of the corresponding pyrroloacridine-1(2H)-one.

**Table 4:** Optimization of the reaction conditions for synthesis of 4,4-dimethyl-2-phenyl-4,5-dihydropyrrolo[2,3,4-kl]acridin-1(2H)-one.

Entry^[a]^	Cat. (mg)	Solvent	Temp (ºC)	Yield^[b]^
1	60	PEG	110	91
2	60	DMF	110	84
3	60	Toluene	110	Trace
5	50	PEG	100	76
6	40	PEG	100	64
7	30	PEG	100	48
^[c]^8	–	PEG	100	N.R
9	60	PEG	80	68
^[d]^10	60	PEG	100	N.R
^[e]^11	60	PEG	100	N.R

^[a]^Reaction conditions:  aniline (1 mmol), dimedone (1 mmol), isatin (1 mmol), Co-DAT‐MOF, Solvent.

^[b]^Isolated yield.

^[c]^When the reaction was conducted in the absence of Co-DAT‐MOF.

^[d, e]^When the reaction was conducted in the presence of Co (NO3)3.6H2O, and 4, 6-Diamino-2-thiopyrimidine.Time. 2.5h.

**Table 5:** The preparation of pyrroloacridine-1(2H)-one derivatives.

Entry^[a]^	Product	Time (h)	Yield^[b]^ (%)	M.P
1		2.5	91	186–187^49^
2		3.5	95	179–182^49^
3		3	94	210–212^49^
4		3	94	200–204^50^
5		2.5	80	195–200
6		2.5	82	218–220^50^
7		1.5	95	130–131^49^
8		2	70	160–162^49^

^[a]^Reaction conditions: amine (1 mmol), dimedone (1 mmol), isatin (1 mmol), Co-DAT‐MOF, solvent.

^[b]^Isolated yield.

**Figure 12:**
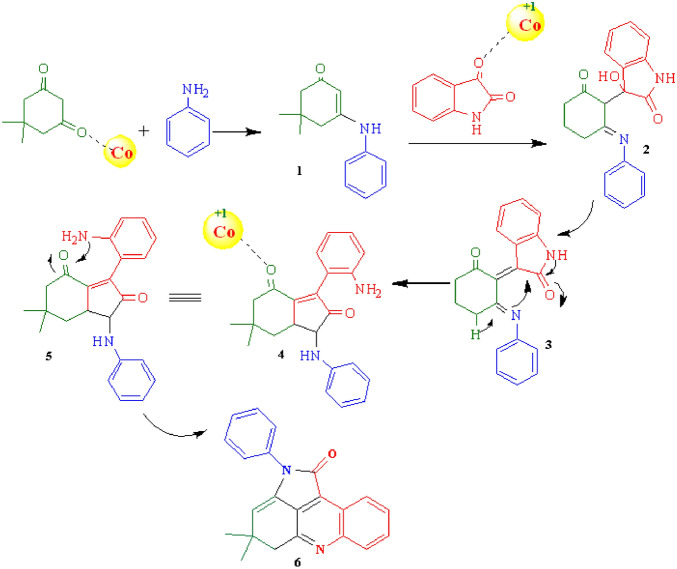
Proposed mechanism for the synthesis of pyrroloacridine -1(2H)-one.

Moreover, a plausible mechanism on the basis of the previous publications for the synthesis of pyrroloacridine-1(2H)-one derivatives has been shown in Fig. 12.

## Heterogeneity studies

### Hot filtration

Hot filtration is another technique to know the heterogeneity of a reaction. Hot filtration technique was carried out for the synthesis 4, 4-dimethyl-2-phenyl-4,5-dihydropyrrolo[2,3,4-kl]acridin-1(2H)-one. The catalyst was separated from the reaction mixture by a simple filtration when the reaction proceeded past 50% completion. We have observed that no further reaction occurred after the separation of the catalyst which means that the Co catalyst remains on the surface during the reaction.

**Figure 13:**
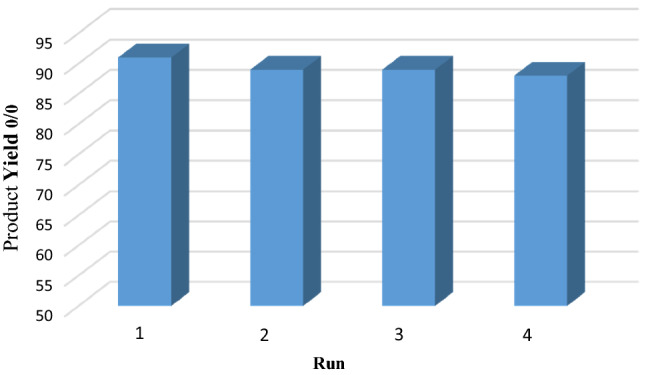
Catalyst recycling study.

One of the most important features of the catalyst is the ability to be recycled. To this aim, the reusability of the mesoporous catalyst has been investigated for the synthesis of 4, 4-dimethyl-2-phenyl-4, 5-dihydropyrrolo [2,3,4-kl] acridin-1(2H)-one using the reaction aniline, dimedone, and isatin. Figure 13 displays that the catalyst could be retrieved by simple filtration and recycled at least 4 times without important loss of its high catalytic activity. Also, the amounts of cobalt leaching after recycling of catalyst was analyzed using ICP-OES. Base on such analysis, the amounts of cobalt in fresh and reused catalyst are 0.065 mol.g^−1^ and 0.061 mol.g^−1^ respectively, which shows that cobalt leaching from Co-DAT‐MOF is very low.

**Figure 14:**
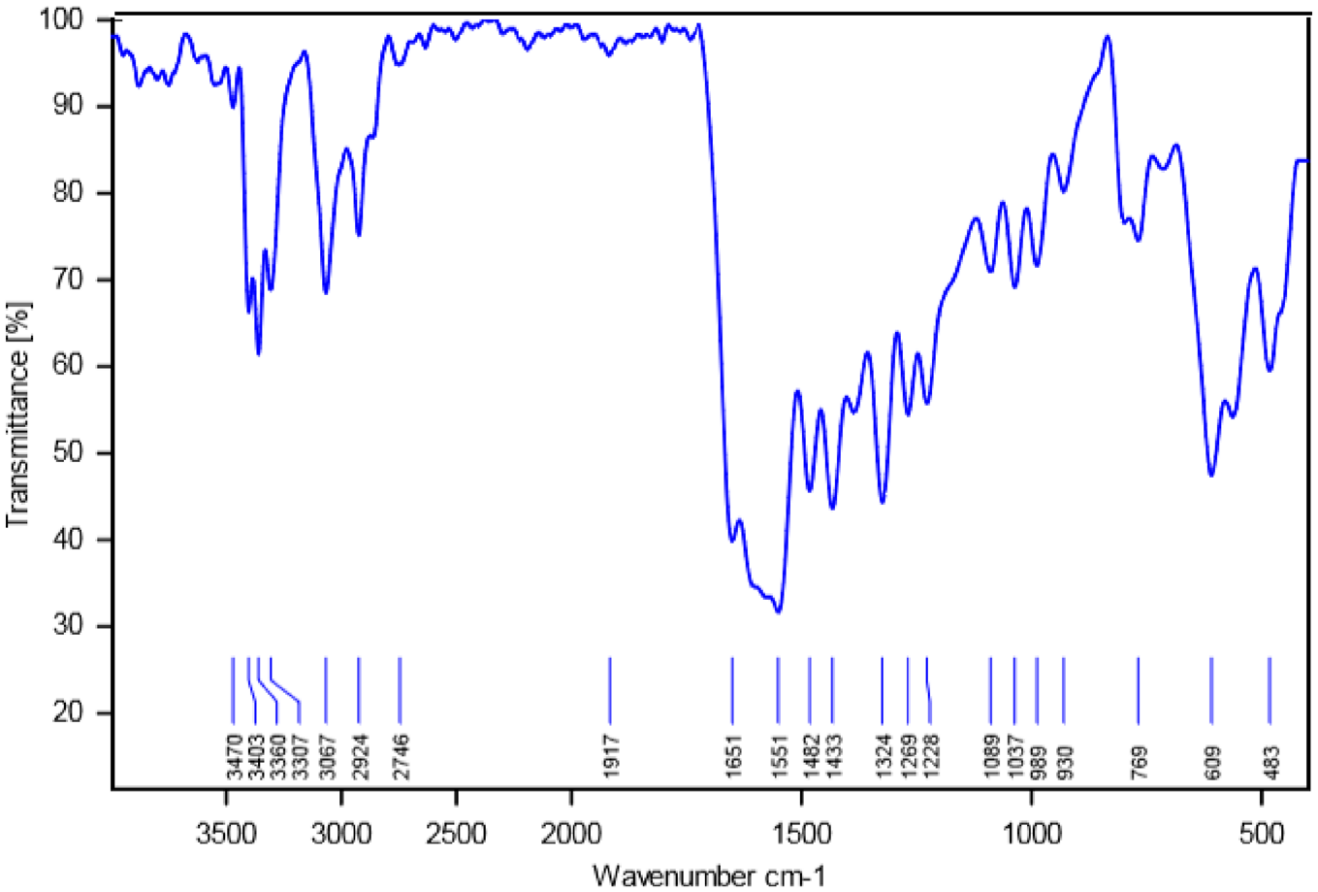
FT-IR spectra of Co-DAT‐MOF after recovery.

The recovered catalyst was analyzed to prove stability and the recoverability using FT-IR, and XRD techniques. The FT-IR spectrum and XRD pattern of the recovered Co-DAT‐MOF indicate that this catalyst can be recycled without any change in its structure (Figs. 4 and 14).

### Comparison of the catalyst

The activity of the prepared catalyst for synthesis of chromeno [2, 3- d] pyrimidin-8-amines was compared with previously reported data in the literature. From Table 6, it is clear that Co-DAT‐MOF worked remarkably well to give the desired product within 60 min in 98% yield in shorter reaction.

**Table 6:** Comparison of Co-DAT‐MOF for the synthesis of 7,10-bis(4-chlorophenyl)-7H-benzo[7,8]chromeno[2,3-d]pyrimidin-8-amine with previously reported procedures.

Enty	Catalyst	Time (min)	Yield	Ref
1	1-butyl-3-methylimidazolium tetrafluoroborate [bmim]BF_4_ ionic liquid	105	88	^47^
2	2-Hydroxyethylammonium formate [2-HEAF] as a mildly basic ionic liquid	45	91	^51^
3	Decorated peptide nanofibers with Cu nanoparticles	150	95	^48^
4	Cobalt complex	120	93	^52^
5	Co-DAT‐MOF	60	98	This work

## Conclusions

The Co-DAT‐MOF was successfully synthesized using a facile solvothermal method and characterized using XRD, FT-IR, TGA, BET, TPD-NH_3_, ICP-EOS, and SEM techniques. The Co-DAT‐MOF particles have a microspheres shape, and good thermal stability. To explore the acidic properties of applied Co-DAT‐MOF, the NH_3_-TPD technique was employed. The peak observed at (95–300 °C) is attributed to weak acid sites, and the peak at range of temperatures (300–475 °C) to strong acidic sites. The N_2_ sorption isotherm shows that Co-DAT‐MOF possesses type IV sorption isotherm. The catalyst was found to be highly efficient and could be reused for four catalytic cycles. This study provides a novel strategy to synthesis of pyrroloacridine-1(2H)-one and chromeno [2, 3-d] pyrimidin-8-amines.

## Supplementary Information

Supplementary Information.

## Data Availability

All data generated or analysed during this study are included in this published article [and its supplementary information files].
