# Supplementary material for: 4,6-Diamino-2-thiopyrimidine-based Cobalt Metal Organic Framework (Co-DAT‐MOF): green, efficient, novel and reusable nanocatalyst for synthesis of multicomponent reactions
Source: Sci Rep. 2023 May 9;13:7502. doi: 10.1038/s41598-023-34001-5 (PMC10169762; doi:10.1038/s41598-023-34001-5)

**4,6-Diamino-2-thiopyrimidine-based Cobalt Metal Organic Framework (Co-DAT‐MOF): green, efficient, novel and reusable nanocatalyst for synthesis of multicomponent reactions**

Arash Ghorbani-Choghamarani,*^a^ Zahra Kakakhani,^b^ Zahra Taherinia^b^

Address correspondence to Prof*.* A. Ghorbani-Choghamarani^a^, Department of Organic Chemistry, Faculty of Chemistry, Bu-Ali Sina University, Hamedan 6517838683, Tel: +988138282807, Fax: +988138380709, Iran; E-mail: [a.ghorbani@basu.ac.ir](mailto:a.ghorbani@basu.ac.ir), [arashghch58@yahoo.com](mailto:arashghch58@yahoo.com). ^b^ Department of Chemistry, Faculty of Science, Ilam University, Ilam, Iran

**Selected apectral data**

**Table 2, entry 1(Product 1a):**^1^H NMR (400 MHz, DMSO) δ ppm:  4.82(s, 1H), 6.86(d, *J*=8Hz, 2H), 7.37-7.32(m, 5H), 7.45-7.41(m, 4H), 7.60 (d, *J*=8, 3H), 7.80(d, 2H), 8.25(s, 2H).

**Table 2, entry 3(Product 1c):**^1^H NMR (400 MHz, DMSO) δ ppm:  2.26(s, 3H), 4.85 (s, 1H), 7.38-7.35(m, 5H), 7.64-7.59(m, 6H), 7.89(d, *J*=8Hz, 3H), 8.36(d, *J*=8Hz, 2H).

**Table 2, entry 9(Product 1i):**^1^H NMR (250 MHz, DMSO) δ ppm:  4.91(s, 1H), 7.18-7.05(m, 7H), 7.58-7.48(m, 6H), 7.88(d, *J*=7.8Hz, 2H), 8.23 (d, *J*=8.2 Hz, 1H).

**Table 4, entry 6(Product 2f):**^1^H NMR (400 MHz, CHCl_3_)8.89 (d, *J* = 8.8 Hz, 1H), 8.73 (d, 1H, *J* = 7.6 Hz, 1H), 7.89-7.84 (m, 2H), 7.46-7.36 (m, 4H), 5.85 (s, 1H), 3.62(s, 2H), 2.45 (s, 2H), 1.58 (s, 6H).


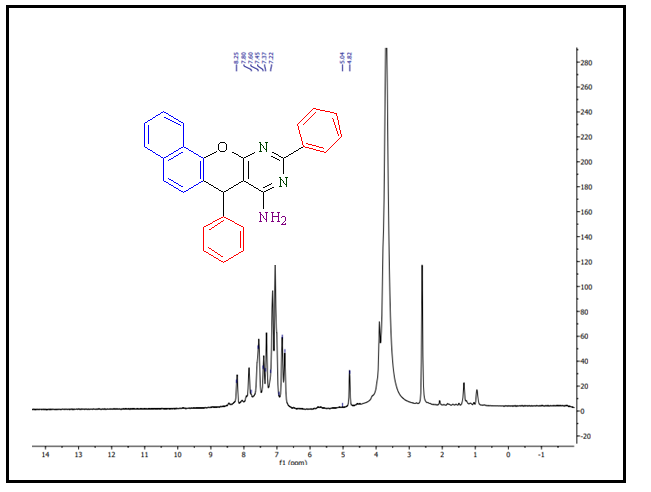


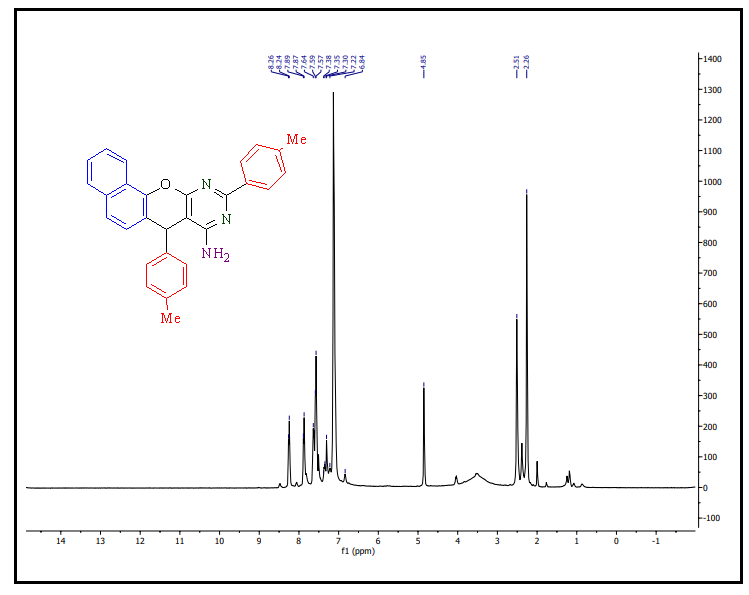


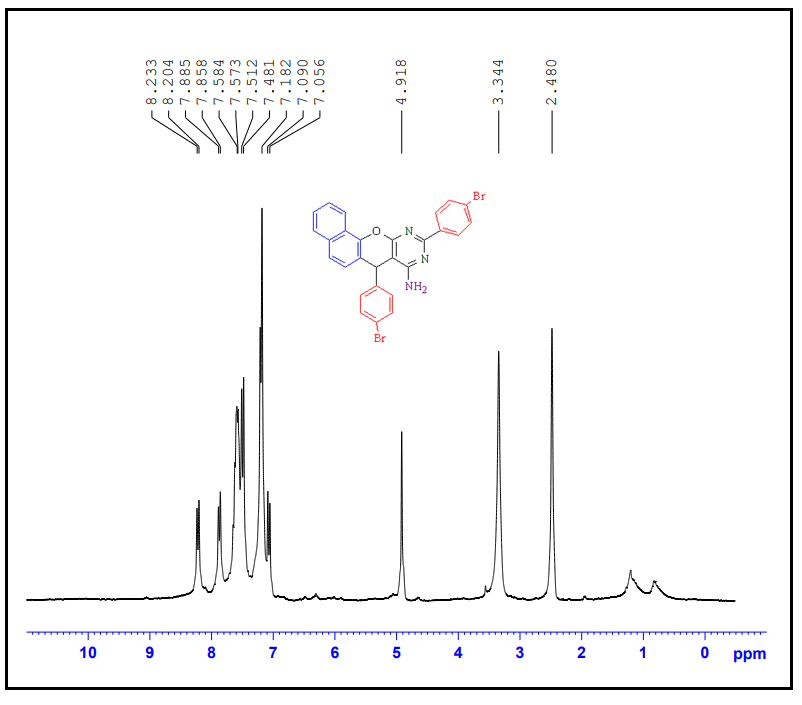


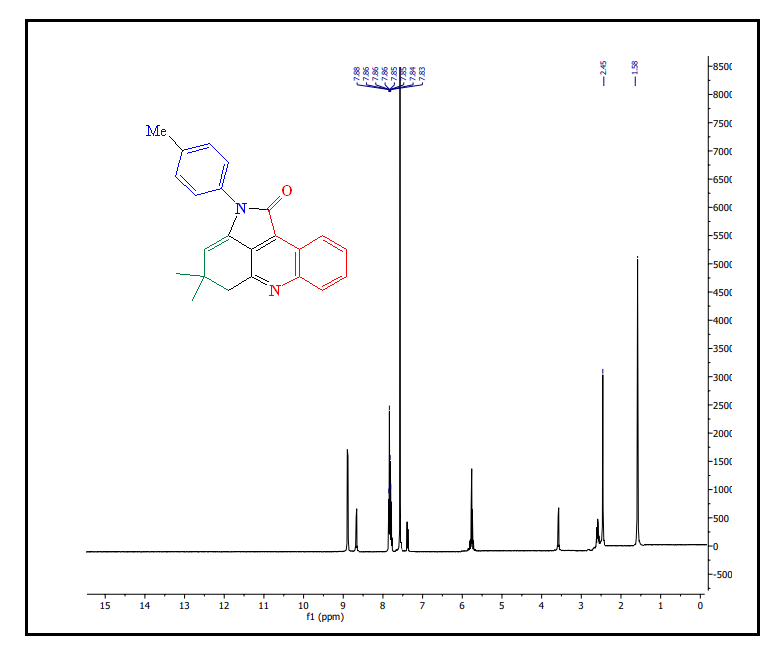

Supplement: Supplementary file 1 — Supplementary Information. [file 41598_2023_34001_MOESM1_ESM.docx]
